# Long non-coding RNA ARAP1-AS1 accelerates cell proliferation and migration in breast cancer through miR-2110/HDAC2/PLIN1 axis

**DOI:** 10.1042/BSR20191764

**Published:** 2020-04-23

**Authors:** Chong Lu, Xiuhua Wang, Xiangwang Zhao, Yue Xin, Chunping Liu

**Affiliations:** Department of Breast and Thyroid Surgery, Union Hospital, Tongji Medical College, Huazhong University of Science and Technology, 1277, Jiefang Avenue, Wuhan 430022, China

**Keywords:** ARAP1-AS1, breast cancer, HDAC2, miR-2110, PLIN1

## Abstract

Breast cancer (BC) poses a great threaten to women health. Numerous evidences suggest the important role of long non-coding RNAs (lncRNAs) in BC development. In the present study, we intended to investigate the role of ARAP1-AS1 in BC progression. First of all, the GEPIA data suggested that ARAP1-AS1 was highly expressed in breast invasive carcinoma (BRAC) tissues compared with the normal breast tissues. Meanwhile, the expression of ARAP1-AS1 was greatly up-regulated in BC cell lines. ARAP1-AS1 knockdown led to repressed proliferation, strengthened apoptosis and blocked migration of BC cells. Moreover, ARAP1-AS1 could boost HDAC2 expression in BC through sponging miR-2110 via a ceRNA mechanism. Of note, the UCSC predicted that HDAC2 was a potential transcriptional regulator of PLIN1, an identified tumor suppressor in BC progression. Moreover, we explained that the repression of HDAC2 on PLIN1 was owing to its deacetylation on PLIN1 promoter. More importantly, depletion of PLIN1 attenuated the mitigation function of ARAP1-AS1 silence on the malignant phenotypes of BC cells. To sum up, ARAP1-AS1 serves a tumor-promoter in BC development through modulating miR-2110/HDAC2/PLIN1 axis, which may help to develop novel effective targets for BC treatment.

## Introduction

Breast cancer (BC) is the one of most prevalent carcinomas that mainly occurs in women worldwide [1,2]. The prognosis of BC in China is still poor and the mortality remains high due to the immature diagnostic and therapeutic strategies, though the incidence is not that high as other high-resource countries [3,4]. Consequently, patients with BC always develop into advanced stages even with distant metastasis, while the metastatic BC is not curable [5]. Though researchers have made great efforts in improving the living quality of BC patients [6], it is also an urgent necessity to figure out more effective targets for BC treatment.

Long non-coding RNAs (lncRNAs) belong to a cluster of transcripts with over 200 nt in length [7]. It is generally believed that the biogenesis of majority lncRNAs has the same characteristics as the protein-coding mRNAs, for example, splicing and polyadenylation [8–10]. In the past decades, lncRNAs have recognized as pivotal regulator of various cytoplasmic and nuclear activities, so as to regulate gene expression at epigenetic, transcriptional, post-transcriptional, translational and post-translational levels [11,12]. Based on such functions, dysregulation of lncRNAs contributes to multiple pathological states, including cancer [13,14]. For example, lncRNA ANCR mitigates BC cell invasion and metastasis by degrading EZH2 [15]. In this research, ARAP1 antisense RNA 1 (ARAP1-AS1) is a novel lncRNA, which has been validated as an oncogene in bladder cancer. ARAP1-AS1 depletion suppressed the proliferation and migration of bladder cancer cells [16], in addition, a latest research illustrated the oncogene role of ARAP1-AS1 in colorectal cancer. ARAP1-AS1 knockdown repressed cell invasion, migration and EMT process in colorectal cancer [17]. According to GEPIA database, the expression of ARAP1-AS1 was aberrantly high expressed in BRC tissues (breast invasive carcinoma) compared with that in corresponding non-tumor tissues. Nevertheless, the function and molecular regulation mechanism of ARAP1-AS1 in BC development has not been reported yet.

Therefore, we aimed to explore whether ARAP1-AS1 acted an oncogene in BC, including the effects of ARAP1-AS1 on BC cell proliferation and migration as well as its molecular mechanism in BC.

## Materials and methods

### Cell lines

Normal human mammary epithelial cells (MCF10A) and the accepted BC cell lines (MDA-MB-468, SKBR3, MCF7 and MDA-MB-231) were purchased from the Shanghai Cell Bank of the Chinese Academy of Sciences (Shanghai, China). All cells were allowed to grow in Dulbecco modified Eagle medium supplemented with 10% (v/v) fetal bovine serum (PAN-Biotech, Aidenbach, Germany), 1% penicillin–streptomycin antibiotics (Beyotime, Shanghai, China) in a humidified atmosphere containing 5% CO_2_ at 37°C. DMEM medium was changed every third day.

### Transfection plasmids

The short hairpin RNAs (shRNAs) against human ARAP1-AS1 (shARAP1-AS1#1/2), against human HDAC2 (shHDAC2#1/2) and against human PLIN1 (shPLIN1) were designed and constructed at GenePharma (Shanghai, China). Non-specific shRNAs were simultaneously obtained and used as control (shCtrl). The aforementioned shRNA plasmids were separately transfected into MCF7 and MDA-MB-231 cells at the final concentration of 20 nm for 48 h using Lipofectamine 2000 (Invitrogen, Carlsbad, CA, U.S.A.). Overexpression of miR-2110 or ARAP1-AS1 in cells was achieved by transfected with either miR-2110 mimics (Genepharma) or pcDNA3.1/ARAP1-AS1 (GeneCopoecia, Guangzhou, China), along with their respective control. Cell transfection was run in triplicate. And related sequences of plasmids were provided in Supplementary Table S1.

### RNA isolation and quantitative real-time polymerase chain reaction (qRT-PCR)

To extract RNA, TRIzol reagent (Invitrogen, Carlsbad, CA, U.S.A.) was utilized in accordance with standard method. Reverse transcribing RNA into cDNA was generated by use of Reverse Transcription Kit (A5001, Promega, Madison, WI, U.S.A.). QRT-PCR was carried out to detect the relative expression using SYBR Premix Ex Taq™ II kit (Takara, Shiga, Japan). GAPDH or U6 served as internal control for lncRNA, mRNA or miRNA. The relative expressions were all calculated using 2^−ΔΔCt^ comparative method. And related primer sequences were provided in Supplementary Table S1.

### Bioinformatics analyses

ARAP1-AS1 expression in human BRCA (breast invasive carcinoma) tissues was found to be higher than that in human normal tissues through GEPIA database (http://gepia.cancer-pku.cn/index.html) [18]. Fold change more than 1.5 and *P*-value less than 0.01 between the tumor and normal tissues were considered as statistically significant. The miRNAs that might bind with ARAP1-AS1 were obtained by utilizing DIANA tools (http://diana.imis.athena-innovation.gr/DianaTools/index.php?r = lncExperimental/index) [19]. The putative binding sites of miR-2110 and ARAP1-AS1 or HDAC2 were predicted by Starbase version 2.0 (http://starbase.sysu.edu.cn/) [20].

### Cell proliferation assays

For detecting cell proliferation, CCK-8 assay was conducted on the basis of the recommendation provided by supplier. In brief, the transfected MCF7 and MDA-MB-231 cells were put into 96-well plates (2000 cells/well) and incubated for 48 h. Then, 10 μl CCK-8 solution was added to every well for 2-h incubation at 37°C. The viability of cells after incubation for 0, 24, 48, 72, 96 h was observed through measuring the absorbance values at the wavelength of 450 nm. With respect to EdU assay, MCF7 and MDA-MB-231 cells under different transfected conditions were seeded in 96-well plates (3000 cells/well). After 48-h incubation, EdU assay was performed in line with manufacturer’s instructions. Cell proliferation was evaluated by calculating percentage of positive stained cells. Click-iT Alexa Fluor 488 Imaging Kit (Molecular Probes Invitrogen, Carlsbad, CA, U.S.A.) was used following the protocol. Assays were both conducted in triplicate.

### TUNEL staining assay

TUNEL assay was conducted to evaluate cell apoptosis via the InSitu Cell Death Detection kit (Roche, Mannheim, Germany). Apoptosis cells were induced by Adriamycin. 4% formaldehyde was used to fix the cells for 25 min at 4°C. About 0.2% TritonX-100 was applied to permeabilize the cells for 5 min. Subsequently, the cells were equilibrated with 100 μl Equilibration buffer, which lasted for 10 min under normal temperature. Cells were labeled with 50 μl TdT reaction mix at 37 °C for 1 h. To count total cells, the counterstain of the nucleus with DAPI fluorescent dye (Beyotime, Shanghai, China) was performed. Images were acquired with fluorescence microscope (Olympus, Tokyo, Japan). Three fields from three independent experiments were chosen at random to calculate the TUNEL-positive cells.

### Cell migration assay

Transwell chambers (24-well; Costar, Boston, MA, U.S.A.) were applied for cell migration assay. Cells in serum-free media were put into upper chambers afterwards, while medium with 10% FBS was put into the bottom chambers. After incubation for 24 h, migratory cells were treated with 4% PFA (Sigma-Aldrich, Burlington, Massachusett, U.S.A.) and 0.5% Crystal Violet. The cell numbers of three replications were counted in five different fields by a microscope.

### Luciferase reporter assay

The reporter plasmids were obtained via inserting ARAP1-AS1 or HDAC2 fragments with wild-type (WT) or mutant (MUT) binding sites of miR-2110 into the pmiR-RB-REPORT™ (Ribobio, China). HEK-293T cells were co-transfected with wild-type or mutant plasmid vectors and different transfection plasmids. At 48 h post-transfection, luciferase activity was assessed by Dual Luciferase Reporter Assay System (Promega, Madison, WI, U.S.A.). For PLIN1 promoter analyses, HEK-293T was co-transfected with the plasmid vector containing PLIN1 promoter, the pRL-TK-Renilla plasmid (Promega) and shHDAC2 or shCtrl.

### RNA immunoprecipitation assay

The Magna RIP RNA-Binding Protein Immunoprecipitation Kit (Millipore, Bedford, MA, U.S.A.) was applied for RNA immunoprecipitation (RIP) assay based on the user guide. The cell lysis were incubated in RIP immunoprecipitation buffer containing magnetic bead conjugated with human anti-Ago2 antibody (Millipore)The protein was digested by proteinase K to obtain the immunoprecipitated RNA. The immunoprecipitated RNA was purified and subjected to qRT-PCR method.

### Western blotting

All samples were lysed in RIPA buffer, separated by sodium dodecyl sulfate-polyacrylamide gel electrophoresis and transferred to nitrocellulose membranes (GE Healthcare Life Science, Little Chalfont, U.K.). About 5% (w/v) non-fat dry milk in Tris-buffered saline containing Tween 20 was used to seal membranes. Then, membranes were cultured all night with specific primary antibodies, including anti-HDAC2 (ab32117; 1/2000, Abcam, Cambridge, U.S.A.), anti-GAPDH (ab22555; 1/1000, Abcam) and anti-PLIN1 (ab3526; 1/1000, Abcam). Horseradish peroxidase-conjugated secondary antibody was incubated at room temperature (25°C) for 1 h. Finally, membranes were visualized via exposure to Immobilon Western Reagents (Millipore, Billerica, MA, U.S.A.).

### Chromatin immunoprecipitation (ChIP) assay

HEK-293T cells were purchased from ATCC (Manassas, VA, U.S.A.) and used for ChIP assay. First, cells were fixed in formaldehyde for 10 min to obtain the DNA–protein cross-links. Thereafter, cell lysates were subjected to ultrasonic to generate 200–500-bp chromatin fragments and immunoprecipitated with HDAC2-specific antibody (Millipore) and IgG as control. Precipitated chromatin of at least three independent experiments was retrieved and analyzed by qRT-PCR method.

### RNA pull-down assay

The biotin-labeled ARAP1-AS1 (sense), as a probe, or biotin-labeled ARAP1-AS1 AS (anti-sense), as negative control (NC), were reversely transcribed via Biotin RNA labeling mix (Roche Diagnostics, U.S.A.) and T7 RNA polymerase (Roche, Switzerland). Then, the samples were processed by RNase-free DNase I (Roche) and then purified using the RNeasy Mini Kit (Qiagen, U.S.A.). MCF7 cells were lysed in RIPA buffer for half an hour and the lysates were mixed with biotin-labeled ARAP1-AS1, and then went through 1-h incubation at 4°C. Next, the reaction mixture was cultured with streptavidin agarose beads (Life Technologies, U.S.A.) for 60 min under normal temperature. QRT-PCR was applied for measuring the co-precipitated RNAs.

### Statistical analysis

Data of at least three independent experiments were shown as the mean ± standard deviation (SD). The software for statistical analysis were GraphPad Prism version 5.0 (GraphPad Software, La Jolla, CA, U.S.A.) and SPSS software (version 13.0; SPSS, Chicago, IL, U.S.A.). The significant differences were analyzed by Student’s *t*-test or one-way analysis of variance. *P*-value less than 0.05 indicated a statistically significant difference.

## Results

### ARAP1-AS1 is overexpressed in BC cell lines and its knockdown hinders BC cell proliferation and migration

**Figure 1:**
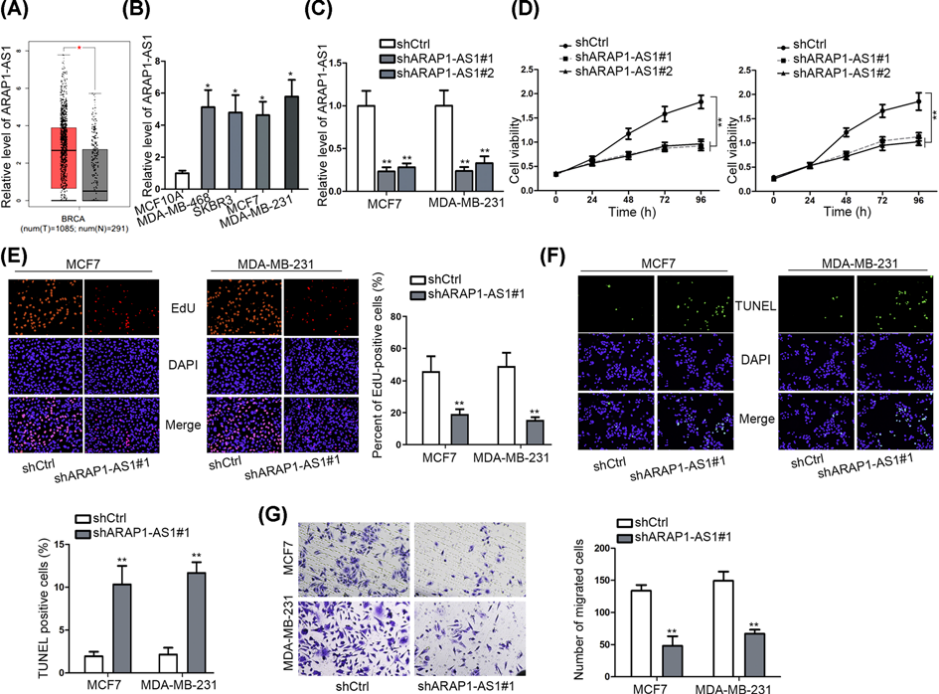
Silencing ARAP1-AS1 suppressed BC cell proliferation and migration (**A**) GEPIA indicated that AEAP1-AS1 was up-regulated in BRCA tissues. (**B**) Relative expression of ARAP1-AS1 in several BC cell lines and the normal MCF-10A cells was tested by qRT-PCR. (**C**) QRT-PCR results of ARAP1-AS1 level in MCF7 and MDA-MB-231 cells in response to the transfection of shRNAs targeting ARAP1-AS1. (**D**) The impact of ARAP1-AS1 silence on the viability of MCF7 and MDA-MB-231 cells was assessed by CCK-8 assay. (**E**) EdU assay was performed to evaluate BC cell proliferation in face of ARAP1-AS1 inhibition. (**F**) Cell apoptosis influenced by ARAP1-AS1 silence was assayed by TUNEL assay. (**G**) The migration ability of BC cells with or without ARAP1-AS1 knockdown was determined by transwell assay; *P <0.05, **P <0.01.

To investigate the potential function of ARAP1-AS1 in BC progression, we first comprehend its aberrantly high expression level in BRCA (breast invasive carcinoma) tissues compared with the normal breast tissues according to GEPIA database (Figure 1A). Meanwhile, ARAP1-AS1 exhibited a higher expression in several BC cell lines (MDA-MB-468, SKBR3, MCF7 and MDA-MB-231) than that in the normal human mammary epithelial cell MCF-10A, and the highest level of ARAP1-AS1 was examined in MCF7 and MDA-MB-231 cells (Figure 1B) that were chosen for loss-of-function assays. The qRT-PCR results represented that the transfection of two shRNAs against ARAP1-AS1 could lead to a noticeable depletion on ARAP1-AS1 expression in both MCF7 and MDA-MB-231 cells (Figure 1C). As a consequence, the viability of above two cells was distinctly hampered in response to either shARPA1-AS1#1 or shARAP1-AS1#2 (Figure 1D). In particular, the shARPA1-AS1#1-transfected cells were further used in subsequent studies owing to better knockdown efficiency of shARPA1-AS1#1. With respect to the results of EdU and TUNEL assays, ARAP1-AS1 knockdown markedly restrained cell proliferation but conversely stimulated cell apoptosis in two BC cells (Figure 1E,F). Moreover, the migratory capacity of MCF7 and MDA-MB-231 cells was weakened when down-regulating ARAP1-AS1 expression (Figure 1G). According to these data, it could be concluded that ARAP1-AS1 exerted oncogenic function in BC through facilitating proliferation and migration.

### ARAP1-AS1 releases HDAC2 expression by sequestering with miR-2110

**Figure 2:**
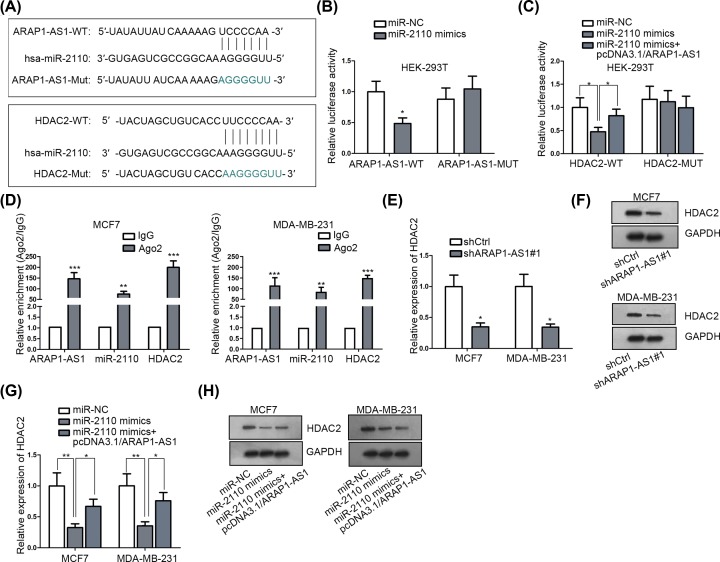
ARAP1-AS1 prompted HDAC2 expression in BC by competitively binding to miR-2110 (**A**) The sequences of wild-type and mutant ARAP1-AS1 and HDAC2 as well as that of miR-2110. (**B** and **C**) Luciferase reporter assays were conducted to confirm the interaction of miR-2110 with both ARAP1-AS1 and HDAC2 at predicted sites. (**D**) The interactions were validated in RISC by RIP assays. (**E–H**) The expression of HDAC2 in indicated cells was examined by qRT-PCR or Western blot, as appropriate. *P <0.05, **P <0.01, ***P <0.001.

Next, we aimed to explore the detailed molecular mechanism whereby ARAP1-AS1 regulated BC progression. Recently, the role of lncRNA as a ceRNA in the development of a variety of human cancers has been recognized increasingly [21]. Here, we also suspected that ARAP1-AS1 might also function in BC through this mechanism. Fortunately, through bioinformatics analysis, as shown in Supplementary Figure S1A, a group of miRNAs that may bind with ARAP1-AS1 were collected through DIANA tools. Then through RNA pull down assay, miR-2110 was the most enriched miRNA in bio-ARAP1-AS1 (sense) group (Supplementary Figure S1B). Similarly, after confirming the binding relation between miR-2110 and ARAP1-AS1/HDAC2, the role of miR-2110 was also elucidated. The pleasing miR-2110 overexpression efficiency was tested (Supplementary Figure S2A). MiR-2110 overexpression obstructed cell proliferation and migration, but stimulated cell apoptosis (Supplementary Figure S2B–E). Furthermore, via Starbase, there were binding sites between miR-2110 and ARAP1-AS1/HDAC2, indicating that miR-2110 as the shared miRNA between ARAP1-AS1 and HDAC2 (Figure 2A). Besides, HDAC2 is an important transcription co-repressor of various tumor suppressor genes [22]. In addition, the tumor-promoter role of HDAC2 in BC was also validated. The knockdown efficiency of HDAC2 was first evaluated (Supplementary Figure S3A). Then several loss-of-function assays were carried out. Cell proliferation and migration were repressed, and apoptosis was encouraged by down-regulating HDAC2 expression (Supplementary Figure S3B–E). Further, the luciferase reporter assays indicated that only miR-2110 mimics, instead of miR-NC, could apparently suppressed the luciferase activity of both ARAP1-AS1-WT and HDAC2-WT, with no influence on that of ARAP1-AS1-Mut and HDAC2-Mut; importantly, the suppression effect of miR-2110 up-regulation on the luciferase activity of HDAC2-WT was overtly attenuated under ARAP1-AS1 overexpression (Figure 2B,C). Subsequently, the co-enrichment of ARAP1-AS1, miR-2110 and HDAC2 was unveiled in the compounds immunoprecipitated by anti-Ago2 (Figure 2D), indicating the interactions among ARAP1-AS1, miR-2110 and HDAC2 in one RNA-induced silencing complex (RISC). Moreover, we explained that both the mRNA and protein levels of HDAC2 were decreased in response to ARAP1-AS1 depletion (Figure 2E,F). Of note, enhanced expression of ARAP1-AS1 remedied the inhibitory function of miR-2110 up-regulation on the HDAC2 expression (Figure 2G,H). Collectively, these findings certified that ARAP1-AS1 acts as a ceRNA of HDAC2 by competitively binding with miR-2110.

### PLIN1 is negatively regulated by HDAC2 at transcriptional level

**Figure 3:**
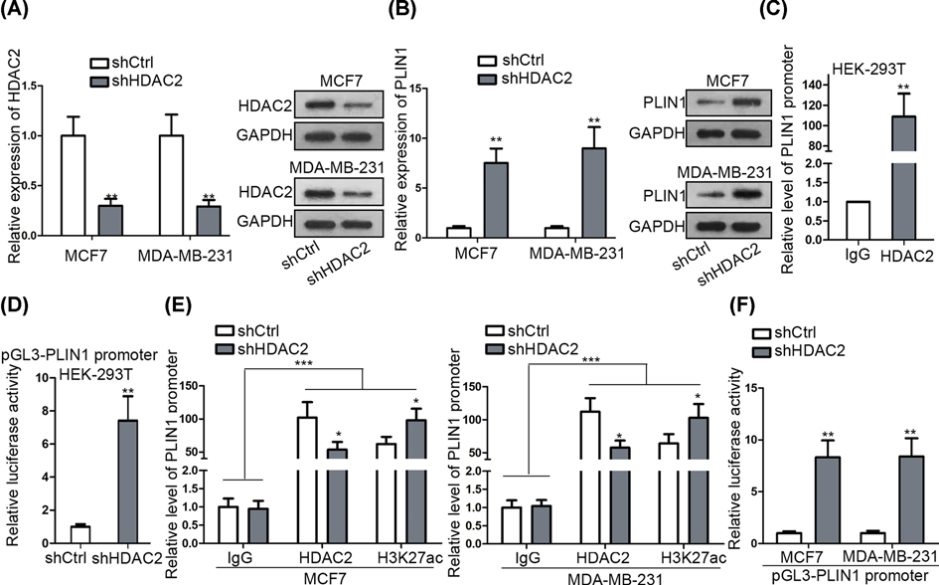
HDAC2 transcriptionally repressed PLIN1 expression in BC (**A** and **B**) The expression level of HDAC2 (A) or PLIN1 (B) in MCF7 and MDA-MB-231 cells with the transfection of shCtrl or shHDAC2 was detected by qRT-PCR and western blot assays. (**C** and **D**) The binding and transcription regulation relations between HDAC2 and PLIN1 promoter was confirmed by ChIP (C) and luciferase reporter assay (D). (**E**) The interaction of HDAC2 protein with PLIN1 promoter and the acetylation state of PLIN1 promoter in BC cells under HDAC2 inhibition were determined by ChIP assay. (**F**) The influence of HDAC2 depletion on PLIN1 transcription in BC cells was estimated by luciferase reporter assay. *P <0.05, **P <0.01, ***P <0.001.

In depth, we explored HDAC2’s potential target that might be involved in ARAP1-AS1-regulated BC development. And PLIN1, a recently identified anti-tumor gene in BC [23,24] was chosen to be research object since UCSC suggested that HDAC2 might be one of potential regulators of it. First of all, we uncovered that inhibition of HDAC2 gave rise to the mRNA and protein expressions of PLIN1 in both MCF7 and MDA-MB-231 cells (Figure 3A,B). In addition, the ChIP assay indicated that HDAC2 could directly bind to PLIN1 promoter, as PLIN1 promoter was considerably concentrated by anti-HDAC2 (Figure 3C). Also, the luciferase activity of PLIN1 promoter was obviously encouraged in HEK-293T cells in face of HDAC2 silence (Figure 3D), further proving that HDAC2 had a negative regulation on PLIN1 transcription. It is well known that HDAC2 functions as a transcriptional co-repressor of its targets through inducing deacetylation of their promoter [25]. Thus, we detected the impact of HDAC2 suppression on the acetylation state of PLIN1 promoter. Consequently, depletion of HDAC2 hindered the interaction of HDAC2 with PLIN1 promoter, and therefore induced a hyper-acetylation on PLIN1 promoter, which eventually contributed to the enhanced PLIN1 transcription in BC cells (Figure 3E,F). Altogether, HDAC2 inhibits PLIN1 expression in BC through highly acetylating the promoter of PLIN1.

### ARAP1-AS1 aggravates BC development by silencing PLIN1

**Figure 4:**
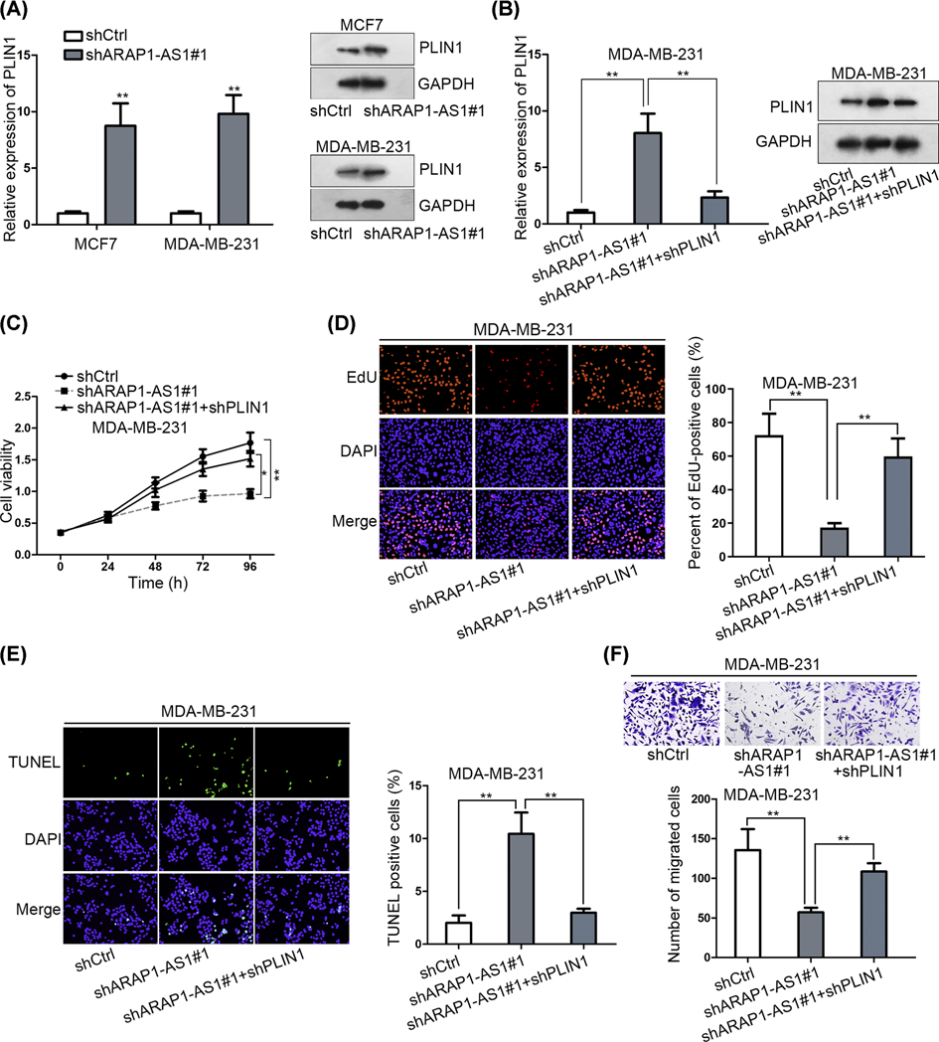
Knockdown of PLIN1 alleviated the inhibitory effect of ARAP1-AS1 on the biological processes of BC cells (**A** and **B**) The results of qRT-PCR and Western blot for the expression of PLIN1 in indicated BC cells. (**C–F**) The impact of PLIN1 depletion on the cellular processes of ARAP1-AS1-silenced MDA-MB-231 cells was evaluated by CCK-8 (C), EdU (D), TUNEL (E) and transwell assays (F). **P <0.01.

In order to confirm whether the contribution of ARAP1-AS1 to BC progression was mediated by a PLIN1-dependent pathway, we first evaluated the effect of ARAP1-AS1 on PLIN1 expression. As anticipated, the mRNA and protein expressions of PLIN1 were prompted in ARAP1-AS1-depleted MCF7 and MDA-MB-231 cells (Figure 4A). However, ARAP1-AS1 suppression-induced PLIN1 expression was normalized in the context of PLIN1 down-regulation (Figure 4B). Intriguingly, the inhibitory impact of ARAP1-AS1 knockdown on the proliferation of BC cells was alleviated after co-transfecting shPLIN1 (Figure 4C,D). Meanwhile, the apoptosis that promoted in ARAP1-AS1-silenced cells was attenuated upon PLIN1 repression (Figure 4E). Also, knockdown of PLIN1 reversed the obstructive function of shARAP1-AS1 on cell migration (Figure 4F). By and large, HDAC2 down-regulated PLIN1 was involved in ARAP1-AS1-facilitated BC progression.

## Discussion

Recently, the emerging role of lncRNAs in multiple cancers including BC has been reported by numerous studies [14]. For example, Kim et al. elucidated that MALAT1 inhibits the metastasis of BC [26]. Niknafs et al. indicated the implication of DSCAM-AS1 in BC progression [27]. And Huang et al. demonstrated that lnc015192 promotes BC metastasis via miR-34a/Adam12 signaling [28]. In the present study, we investigated the role of a novel lncRNA ARAP1-AS1, which was suggested to be up-regulated in BRCA (breast invasive carcinoma) tissues by GEPIA In the present study, we revealed that ARAP1-AS1 exerted a pro-tumor function in BC, evidenced by cell proliferation and migration was restrained by ARAP1-AS1 knockdown, in line with its tumorigenic role previously identified in bladder cancer [16].

As is well-known, lncRNAs can modulate gene expressions through diverse ways though they lack of the ability of encoding proteins [29]. And it has extensively reported that of lncRNAs serve as ceRNAs to modulate mRNA of certain protein-coding genes via sponging miRNAs in recent years [30,31]. As an example, UICLM promotes the metastasis of colorectal cancer by serving as a ceRNA of ZEB2 via interacting with microRNA-215 [32]. LINC01234 acts as a ceRNA to modulate CBFB expression in gastric cancer by absorbing miR-204-5p [33]. Herein, we uncovered a ceRNA mechanism of ARAP1-AS1-miR-2110-HDAC2 in BC cells, among which miR-2110 has been identified as an onco-suppressor in in neuroblastoma [34] and HDAC2 is a well-recognized transcriptional co-repressor that plays a carcinogenic role in BC progression [35–37]. In our research, miR-2110 overexpression inhibited cell proliferation, migration and stimulated cell apoptosis in BC. Nevertheless, HDAC2 knockdown led to the opposite results. These findings proved the tumor inhibitor role of miR-2110 as well as the tumor-facilitator role of HDAC2 in BC.

Further, we also explored the potential downstream effector of ARAP1-AS1/miR-2110/HDAC2 axis in the present study. PLIN1 is a prognostic predictor for BC patients that was firstly uncovered in 2015 [38], while its prognostic significance and suppressive role in BC was further elucidated by Zhou et al. in 2016 [39]. Currently, we validated that PLIN1 could be transcriptional silenced by HDAC2 due to the deacetylation of HDAC2 on PLIN1 promoter. Furthermore, the negative regulation of ARAP1-AS1 on PLIN1 expression was confirmed. Moreover, the implication of PLIN1 in ARAP1-AS1-contributed BC development was also proved here, according to the rescuing role of PLIN1 silencing on sh-ARAP1-AS1 downregulation-mediated cell proliferation and migration.

All in all, our study unmasked a novel potential mechanism of ARAP1-AS1/miR-2110/HDAC2/PLIN1 axis underlying the pathogenesis and development of BC, which may provide new targets for the diagnosis and treatment of BC.

## Supplementary Material

Supplementary Figures S1-S3

Supplementary Table S1

## Abbreviations

BC: breast cancer
BRAC: breast invasive carcinoma
lncRNA: long non-coding RNA
qRT-PCR: quantitative real-time polymerase chain reaction
