# Supplementary material for: Long non-coding RNA ARAP1-AS1 accelerates cell proliferation and migration in breast cancer through miR-2110/HDAC2/PLIN1 axis
Source: Biosci Rep. 2020 Apr 23;40(4):BSR20191764. doi: 10.1042/BSR20191764 (PMC7197975; doi:10.1042/BSR20191764)
Supplement: Supplementary Figures S1-S3 [file BSR-2019-1764_supp.pdf]

A

Gene	miRNA	Score	DIANA Links
ARAP1-AS1	hsa-miR-365a-5p	0.929	mT TB InE mP
ARAP1-AS1	hsa-miR-6810-5p	0.889	mT TB InE mP
ARAP1-AS1	hsa-miR-4652-5p	0.881	mT TB InE mP
ARAP1-AS1	hsa-miR-365b-5p	0.878	mT TB InE mP
ARAP1-AS1	hsa-miR-3191-3p	0.858	mT TB InE mP
ARAP1-AS1	hsa-miR-4735-3p	0.848	mT TB InE mP
ARAP1-AS1	hsa-miR-2110	0.823	mT TB InE mP
ARAP1-AS1	hsa-miR-450a-2-3p	0.820	mT TB InE mP
ARAP1-AS1	hsa-miR-6857-5p	0.820	mT TB InE mP
ARAP1-AS1	hsa-miR-4446-3p	0.745	mT TB InE mP
ARAP1-AS1	hsa-miR-149-3p	0.727	mT TB InE mP
ARAP1-AS1	hsa-miR-4663	0.722	mT TB InE mP
ARAP1-AS1	hsa-miR-6770-5p	0.704	mT TB InE mP

B

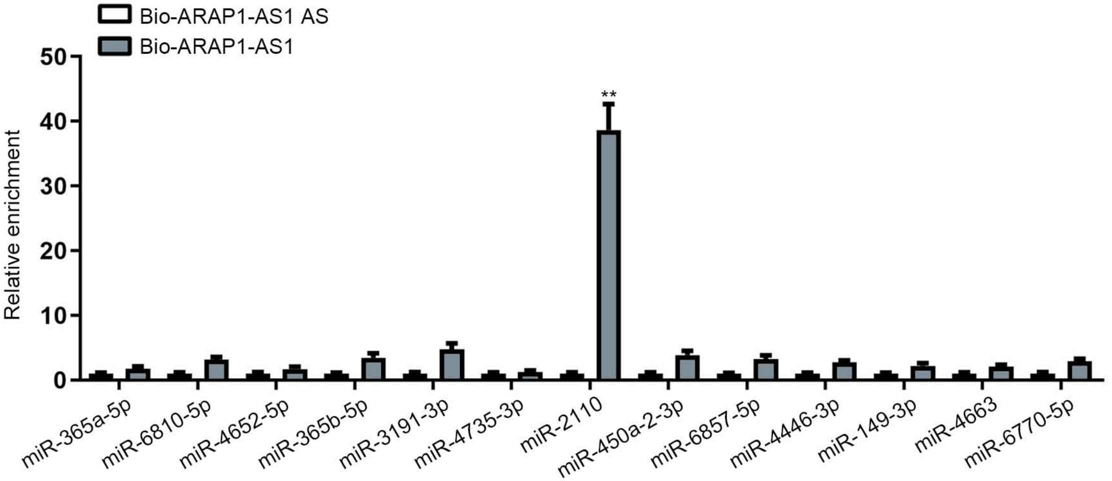

Supplementary Figure 1 (A) A group of miRNAs that might bind with ARAP1-AS1were obtained through DIANA Tools. (B) RNA pull down assay detected the enrichment of the miRNAs in bio-ARAP1-AS1 (sense) and bio-ARAP1-AS1 AS (anti-sense) groups. ** P<0.01.

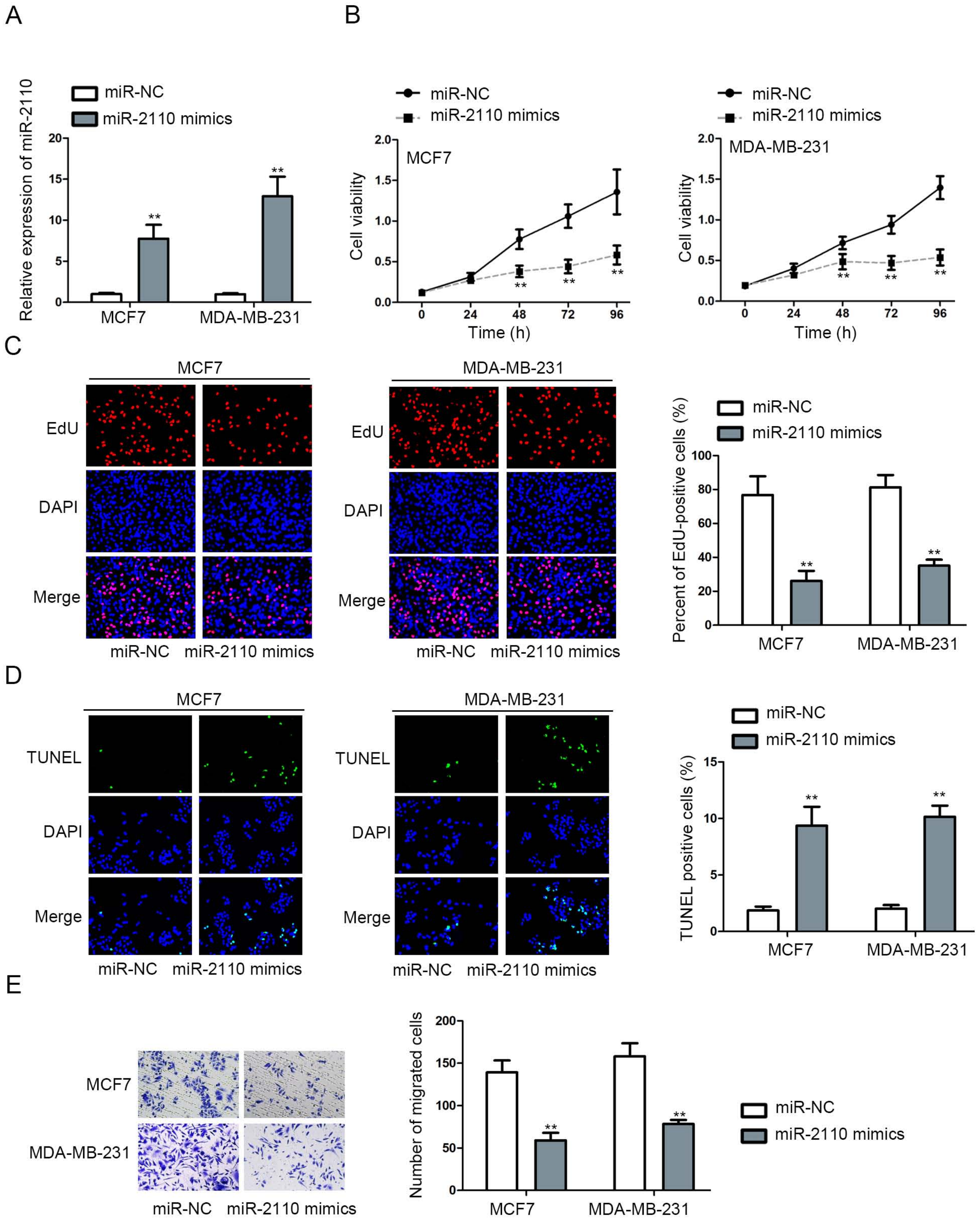

A
B
C
D
E
miR-NC
miR-2110 mimics
Relative expression of miR-2110
MCF7
MDA-MB-231
Cell viability
Time (h)
EdU
DAPI
Merge
Percent of EdU-positive cells (%)
TUNEL
TUNEL positive cells (%)
Number of migrated cells

Supplementary Figure 2 (A)The overexpression efficiency of miR-2110 was evaluated by qRT-PCR. (B-C) BC cell viability and proliferation abilities were measured by CCK-8 andEdUassays. (D) BC cell apoptosis was tested by TUNEL assay. (E) BC cell migration ability was detected by transwell assay. ** P<0.01.

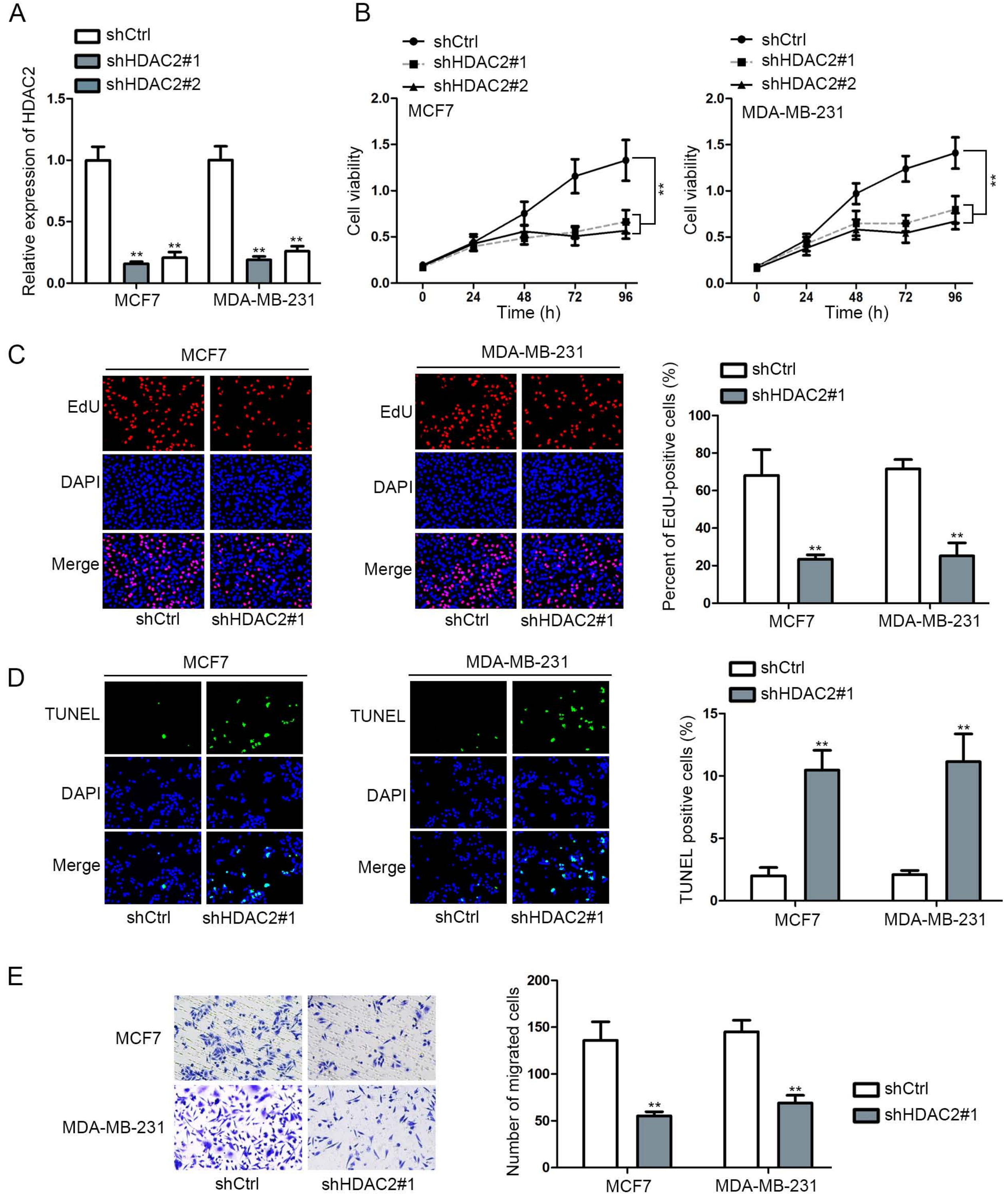

A
shCtrl
shHDAC2#1
shHDAC2#2
Relative expression of HDAC2
1.5
1.0
0.5
0.0
**
MCF7
MDA-MB-231
B
MCF7
MDA-MB-231
Cell viability
2.0
1.5
1.0
0.5
0.0
0
24
48
72
96
Time (h)
C
EdU
DAPI
Merge
Percent of EdU-positive cells (%)
100
80
60
40
20
0
D
TUNEL
TUNEL positive cells (%)
15
10
5
E
Number of migrated cells
200
150
100
50

Supplementary Figure 3 (A)The knockdown efficiency of HDAC2 was evaluated by qRT-PCR. (B-C) BC cell viability and proliferation abilities were measured by CCK-8 andEdUassays. (D) BC cell apoptosis was tested by TUNEL assay. (E) BC cell migration ability was detected by transwell assay. ** $P<0.01$.
